# Complete Genome Sequence of a Zika Virus Strain Isolated from the Serum of an Infected Patient in Thailand in 2006

**DOI:** 10.1128/genomeA.00121-18

**Published:** 2018-03-08

**Authors:** Narong Nitatpattana, Kumchol Chaiyo, Supoth Rajakam, Kanya Poolam, Kusuma Chansiprasert, Norapath Pesirikan, Supanat Buree, Ekkarat Rodpai, Sutee Yoksan

**Affiliations:** a Institute of Molecular Biosciences, Mahidol University, Nakhon Pathom, Thailand; b Government Pharmaceutical Organization Ministry of Public Health, Bangkok, Thailand

## Abstract

The complete genome of Zika virus (ZIKV) strain CVD_06-274 was isolated from the serum of an infected patient in Thailand in 2006. Phylogenetic analysis showed that this strain belongs to the Asian lineage and also high titers in Vero cells (RCB 10-87). It has potential for development as an inactivated ZIKV vaccine.

## GENOME ANNOUNCEMENT

Zika virus (ZIKV) is an icosahedral enveloped arbovirus. ZIKV is a member of the genus Flavivirus, family Flaviviridae. This family also includes dengue virus (DENV), West Nile virus (WNV), and yellow fever virus (YFV) (1). The ZIKV genome is a single-stranded positive-sense RNA approximately 11 kb in length. The viral genome consists of a single open reading frame (ORF) flanked with 5′- and 3′-untranslated regions (UTRs). The ORF encodes 3 structural proteins and 7 nonstructural proteins (2). The structural proteins play a role in viral particle assembly and pathogenicity. The nonstructural proteins and UTRs play a role in viral replication (3). This phylogenetic relationship indicated that ZIKV has 2 major lineages, African and Asian. The Asian-lineage ZIKV was reported in an outbreak in the South Pacific islands, South America, and Asia during 2013 to 2017 (2).

We report the complete genome sequence of ZIKV strain CVD_06-274, isolated from the serum of an infected patient in Thailand in 2006; this study was approved by the Siriraj Ethics Committee, Mahidol University (Si 180/2006). The serum sample was inoculated into Toxorhynchites splendens mosquitoes for 3 passages (4), followed by WHO-certified Vero cells (RCB 10-87) for 3 passages. The infected culture supernatant was positive for flavivirus (5) and ZIKV (6) by real-time quantitative reverse transcription-PCR (qRT-PCR) using the Kapa Probe Fast one-step qRT-PCR kit (Kapa Biosystems, USA) on a Chromo4 system (Bio-Rad, USA). The CVD_06-274 isolate was titrated by plaque assay in an LLC-MK2 cell (7).

Viral RNA was extracted using the viral RNA minikit (Qiagen, Germany) and converted to cDNA using the Maxima H Minus first-strand cDNA synthesis kit (Thermo Scientific, USA). cDNA was amplified using the Phusion Flash high-fidelity PCR master mix (Thermo Scientific). A set of ZIKV whole-genome sequencing primers (8) was used. PCR products were sequenced using the Sanger method (1st Base, Malaysia). Sequences were analyzed using BioEdit 7.0.5 software (9) and the Basic Local Alignment Search Tool (BLAST; https://www.ncbi.nlm.nih.gov/BLAST). The whole-genome sequence of the CVD_06-274 isolate and other ZIKV strains from GenBank were used for phylogenetic tree analysis. A neighbor-joining tree was constructed with 1,000 bootstrap replicates using MEGA6 (10).

We obtained the genome sequence of CVD_06-274, with a total length of 10,623 nucleotides. BLAST analysis showed that the highest identity value, at 99.3%, was to Zika virus/H.sapiens-tc/KHM/2010/FSS13025 strain (GenBank accession number KU955593, Cambodia, 2010) and SK403/13AS strain (GenBank accession number KX051561, Thailand, 2013). A phylogenetic tree revealed that the CVD_06-274 isolate belongs to the Asian lineage. CVD_06-274 produces a medium plaque size (2 to 3 mm) in LLC-MK2 cells. This virus showed a high level of replication at a peak titer of 8 log_10_ PFU/ml in WHO-certified Vero cells, on a par with inactivated-ZIKV vaccine strain PRVABC59 (GenBank accession number KU501215), which was developed by the Walter Reed Army Institute of Research (WRAIR, USA) (11). An inactivated vaccine has high safety and less reactogenicity, and a high viral titer is required to achieve sufficient immunogenicity. The high level of replication of CVD_06-274 in a vaccine production cell line indicated a potential for further development into an inactivated Zika vaccine.

### Accession number(s).

The assembled complete genome sequence of the Zika virus CVD_06-274 was submitted to GenBank under the accession number MG645981.
